# Septic Shock and Bacteremia Secondary to Herbaspirillum huttiense: A Case Report and Review of Literature

**DOI:** 10.7759/cureus.36155

**Published:** 2023-03-14

**Authors:** Ariel Ruiz de Villa, Akankcha Alok, Anuoluwa E Oyetoran, Stephanie P Fabara

**Affiliations:** 1 Internal Medicine, Hospital Corporation of America (HCA) & University of Central Florida (UCF) College of Medicine, North Florida Regional Medical Center, Gainesville, USA

**Keywords:** atypical pneumonia, pneumonia, septic shock, herbaspirillum huttiense, gram-negative bacteremia

## Abstract

The* Herbaspirillum *species are gram-negative bacteria that inhabit soil and water. Infections caused by this pathogen are an uncommon clinical entity. We describe a rare case of septic shock and bacteremia caused by *Herbaspirillum huttiense* in an immunocompetent adult female. The patient, a 59-year-old female, presented to the hospital with circulatory shock, fever, chills, and cough. Chest x-ray revealed right lower lobe lung consolidation consistent with pneumonia, and blood cultures with a positive concerning gram-negative curved rod which was later identified as *H. huttiense*. The patient was treated in the ICU for three days with cefepime and vasoactive agents. After improvement and an additional seven days of hospitalization, the patient was discharged home with a five-day course of oral levofloxacin. Although our patient responded well to cefepime and levofloxacin, meropenem and piperacillin-tazobactam were found to be the most commonly used and the most effective antibiotics to treat *H. huttiense* infections in other reported cases. This is amongst the few reported cases of *H. huttiense* bacteremia in an immunocompetent individual with pneumonia.

## Introduction

*Herbaspirillum species* are gram-negative rod-shaped members of the Betaproteobacteria class [1]. Most species are nitrogen-fixing soil and plant bacteria commonly found in the roots and stems of maize, rice, beans, bananas, sugar cane, and pineapple, as well as in groundwater and drinking water distribution systems [1]. *Herbaspirillum huttiense* is a rare environmental organism and is an uncommon cause of bacteremia in immunocompetent patients, with only a few reported cases in the literature. As a pathogen, it is also exceedingly rare [1].

In our case report, we present an immunocompetent patient affected by this bacteria resulting in septic shock with a most likely route of infection through inhalation or ingestion of contaminated material while caring for an artificial duck pond. We describe our management, approach to diagnosis and treatment, and a literature review of the topic. This case highlights the importance of considering uncommon pathogens in the differential diagnosis of infectious diseases, even in immunocompetent patients.

## Case presentation

Our patient was a 59-year-old female with a past medical history significant for mild COPD (GOLD stage 1), hypertension, tobacco use, and controlled type 2 diabetes mellitus (not insulin-dependent) who presented to the emergency department with a three-day history of fever, chills, and cough. She reported no recent travel, sick contacts, or unusual dietary habits. No recent hospitalizations nor corticosteroid use. No autoimmune diseases and negative HIV status. The patient denied the use of alcohol or recreational drugs.

**Figure 1:**
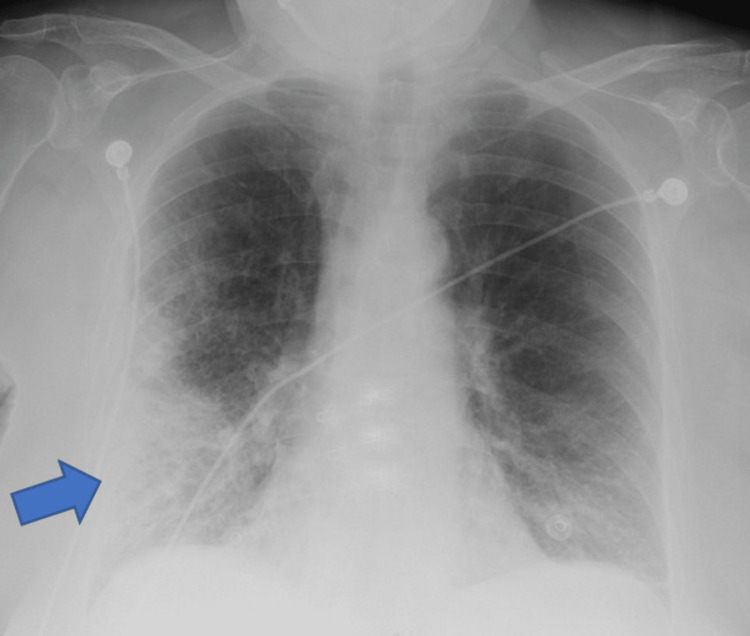
Patient's admission chest X-ray. Blue arrow pointing at pneumonia in the right lower lobe

On arrival, the patient was febrile with a temperature of 100.8°F, tachycardic with a heart rate of 115 beats per minute, and hypotensive with a blood pressure of 80/55 mmHg. Physical examination revealed an alert and oriented patient with dry mucous membranes, decreased skin turgor, and poor peripheral perfusion. There were no significant findings on cardiac examination besides tachycardia; however, lung examination revealed coarse bilateral breath sounds and right lower lobe crackles. Laboratory studies showed leukocytosis (white blood cell count 21,900/μL) which was neutrophilic dominant, without any other relevant abnormalities. Blood cultures were drawn, and the patient was started on empiric broad-spectrum antibiotics, vancomycin and cefepime, 2 liters bolus and continuous intravenous fluids, and continuous norepinephrine infusion titrated to achieve a mean arterial pressure of more than 60 mmHg. The patient was admitted to the intensive care unit (ICU) for further management. Chest X-ray demonstrated right lower lobe opacification concerning an infectious process (Figure 1).

On hospital day 2, blood cultures grew gram-negative curved rods. The patient was then transitioned to cefepime treatment only while receiving blood pressure support, nebulized treatments, and oxygen supplementation. The patient’s clinical condition improved over the next day with the resolution of fever and improvement of both hemodynamic and respiratory status. 

The blood cultures speciated as *Herbaspirillum huttiense*. The patient was diagnosed with septic shock and bacteremia secondary to *H. huttiense.* The infectious disease specialist continued a seven-day course of cefepime before discharging and changing the antibiotic therapy to oral levofloxacin at a dose of 500mg daily for an additional five days. The treatment with cefepime was guided by clinical judgment from the specialist and the overall gram-negative coverage and effectiveness of this antibiotic. Antimicrobial sensitivities were not available until the transition to the oral regimen.

It was revealed that the patient had an artificial duck pond at home, which she thoroughly cleaned approximately one week before the presentation, as the likely source of the initial respiratory infection. She was discharged home in stable condition.

## Discussion

*Herbaspirillium huttiense* is an aerobic, non-fermenting, gram-negative bacillus that thrives in in natural environments and occasionally causes opportunistic infections in humans [2]. Although several species of *Herbaspirillum* have been described in the literature, our discussion focuses primarily on *H. huttiense*, the species isolated in our patient. This organism is comparatively rare, and unlike other species of *Herbaspirillum*, it has been found to cause fatal infections even in immunocompetent hosts. Our case adds to the handful of cases reported in the literature describing life-threatening infections caused by this organism. A detailed review of the literature was done using the PUBMED search engine. Table 1 summarizes our findings and compares the various presentations of *H. huttiense* infection and the treatment guidelines used.

**Table 1:** Table outlining the pertinent cases in the literature. Describing patient's ages, presentation and antibiotic used for treatment. M=male F=female

Manuscript	Age/gender	Presentation	Antibiotic used
Our case	59/F	Pneumonia >septic shock	Cefepime and levofloxacin
First Study of Bacteremia Caused by *Herbaspirillum huttiense* in China: A Brief Research Report and Literature Review. Li et al., 2022 [2]	72/M	Bacteremia	Meropenem and tigecycline> Moxifloxacin and piperacillin-tazobactam
A Case of Infective Endocarditis Due to *Herbaspirillum Huttiense* in a Pediatric Oncology Patient. Gungor et al., 2020 [3]	11/F	Infective endocarditis in pediatric patient	Teicoplanin and piperacillin/tazobactam>meropenem and amikacin.
Septicemia Caused by *Herbaspirillum huttiense* Secondary to Pneumonia. Liu et al., 2019 [4]	93/M	Septicemia	Meropenem and colistin> ceftazidime, minocycline, and trimethoprim/sulfamethoxazole
*Herbaspirillum huttiense *pneumonia in a patient with essential thrombocythaemia. Berardino et al., 2018 [5]	59/F	Pneumonia in the setting of Essential thrombocythemia	Piperacillin-tazobactam
Severe Community-Acquired Pneumonia with Bacteremia Caused by *Herbaspirillum aquaticum *or *Herbaspirillum huttiense* in an Immune-Competent Adult. Regunath et al., 2015 [6]	46/M	Acute respiratory failure	Doxycycline and piperacillin-tazobactam

Most cases described above were found in adults with no particular age predilection, except one case of infective endocarditis found in a pediatric patient (an 11-year-old girl) [3]. There was no specific association with gender in infected patients. The cases described in the table were found in immunocompetent patients, highlighting the virulence of this organism and its ability to colonize and infect presumably healthy hosts. The immune status of the patients described by Berardino et al. and Gungor et al. was questionable, who presented *H. huttiense* infections in patients with a malignancy and a myeloproliferative disorder [2,5]. 

*H. huttiense* predominantly inhabits natural environments, especially water bodies like ponds and lakes. Our patient mentioned a specific history of "cleaning a pond with ducks" before acquiring this infection, reiterating this organism's characteristic feature. Similarly, the patient in the case published by Regunath et al. [6] provided a similar history of ‘fishing’. Another unique trait of this organism is the inability to isolate it in regular blood cultures. Hence, to be able to extract and speciate Herbaspirillum, a special assay called matrix-assisted laser desorption ionizing-time-of-flight mass spectrometry (MALDI-TOF MS), and 16S rRNA sequencing and Next-Generation Sequencing (NGS) has to be used [2]. A similar technique was used to identify this organism in all the cases described above, including ours. In terms of management, although our patient responded well to cefepime and levofloxacin, meropenem and piperacillin-tazobactam were found to be the most commonly used and the most effective antibiotics to treat *H. huttiense* infections in other reported cases.

## Conclusions

*Herbaspirillum Huttiense* is a rare cause of bacteremia and sepsis, and its clinical significance is not well established. This case report features a unique and rare presentation of a gram-negative bacilli bacteremia and septic shock caused by *H. huttiense*, along with a review of literature. With the help of this manuscript, we aim to create awareness and educate clinicians including internists and infectious disease specialists to timely and efficiently diagnose and treat fatal infections caused by *H. huttiense*. This in turn will prevent life-threatening complications and potentially enhance patient care.
